# First report of *Kingella kingae* infection in a paediatric population in Accra, Ghana

**DOI:** 10.11604/pamj.2022.41.95.29528

**Published:** 2022-02-02

**Authors:** Charles Addoquaye Brown, Deborah Abban, Prince Pappoe-Ashong, Alexander Martin-Odoom

**Affiliations:** 1 School of Biomedical and Allied Health Sciences, College of Health Sciences, University of Ghana, Legon, Ghana

**Keywords:** *Kingella kingae*, children, Ghana, bacteremia, blood culture, polymerase chain reaction

## Abstract

**Introduction:**

Kingella kingae is recognized as a frequent source of childhood bacteremia and the commonest agent of skeletal system infections in children 6 months - 4 years old. Several factors, including difficulty in detecting this fastidious organism in routine laboratory assays, result in underdiagnosis of the infections. Species-specific nucleic acid amplification assays, however, significantly improve the detection of K. kingae in blood samples. The aim of this study was to detect K. kingae infection in young children in Accra, Ghana.

**Methods:**

a cross-sectional based study was carried out in three hospitals in Accra. Children with febrile illness and directed by a clinician for blood culture were recruited. Blood samples collected were analysed by culture and polymerase chain reaction (PCR), using universal prokaryotic and K. kingae rtxA primers.

**Results:**

blood samples from 232 children (mean age 20.10 ± 12.57 months) were analysed. Bacteremia (72.4%) was the highest clinical diagnosis particularly in the 12-24 months age group. Only 7 (3.1%) samples showed bacterial growth and were negative for Kingella. PCR with universal prokaryotic primers succeeded in 223 (96.1%) out of 232 samples. PCR with K. kingae rtxA toxin primers was positive for 12 (5.4%) samples, all diagnosed as bacteremia, out of the 223 samples. Eleven (91.7%) out of the 12 K. kingae PCR positives were culture-negative.

**Conclusion:**

Kingella kingae was detected only by PCR specific for the K. kingae rtxA toxin. Kingella kingae may be a potential cause of bacteremia and hence febrile illness in young children living in Accra, Ghana.

## Introduction

*Kingella kingae* is a fastidious Gram-negative coccobacillus and a normal flora of the oropharynx in young children, with a prevalence rate of 10-12% among 2 - 24-month-old children [1]. *Kingella kingae* is recognized as a pathogen responsible for musculoskeletal infections in the paediatric population [2, 3] and responsible for reported outbreaks of invasive *K. kingae* infections in the United States, Israel, and France in toddlers attending child care centres [4-8]. It is also a cause of bacterial endocarditis in children and adults [9]. Most patients with invasive *K. kingae* disease have moderate fever, whiles some are afebrile. Constitutional symptoms are also typically lacking, except in patients with endocarditis [9].

Detection of this fastidious organism strongly depends on the microbiologic methodology used. *Kingella kingae* is difficult to detect in routine laboratory assays because the fastidious nature of the bacterium makes it difficult to grow on solid medium. Also, recovery of *K. kingae* by culture remains unsatisfactory because *K. kingae* from specimen seeded onto solid culture media often results in a lack of growth [10] and thus many illnesses caused by this bacterium are probably overlooked. Results of cultures of bone aspirates and synovial fluid are often negative [11], and even if a bacterium is isolated, complete identification and antibiotic susceptibility testing may require 2 - 3 extra days. Furthermore, clinical presentation of disease due to *K. kingae* is often mild, thus the illness goes under diagnosed which contributes to a delay in diagnosis. This implies that, making *K. kingae* diagnosis requires a high index of suspicion [9].

Improvements in culture techniques and molecular detection methods have led to findings indicating that, particularly in infants and young children, *K. kingae* is a significantly more important pathogen than previously thought [3]. Detection of the organism approaches 100% when polymerase chain reaction (PCR) techniques are used [10]. In a comparative study by Rosey, Abachin [12], inoculation of blood culture vials and seeding samples of synovial fluid onto sheep blood agar and chocolate agar resulted in the isolation of *K. kingae* in 6 out of 94 (6.4%) children, whereas a combination of conventional and real-time PCR with broad-spectrum primers disclosed 15 additional cases. These results clearly demonstrated the superiority of the molecular methods and indicated that a substantial proportion of culture-negative paediatric arthritis may be attributed to *K. kingae* (Rosey *et al*., 2007). Additional studies published over the previous decade have proven that PCR enhances detection of *K. kingae* in samples compared with routine cultures and blood culture vials and indicated that a substantial proportion of culture-negative paediatric arthritis may be attributed to *K. kingae* [2, 13].

Although *K. kingae* is the leading agent of bone and joint infections in early childhood, the vast majority of publications on *K. kingae* infections have originated in countries in the developed world; reports from the developing world are still scarce. Most of the existing cases of *K. kingae* infection and carriage have been studied in Europe, North and South America, Australia [14], Israel [4, 15], New Zealand [16], Japan [17] and, until recently [18], none from Africa. There is not yet widespread knowledge among the medical community of *K. kingae* in Ghana. However, because of the potential risk for severe complications and long-term functional disability of septic arthritis and osteomyelitis in childhood, prompt laboratory confirmation and early administration of effective antimicrobial therapy are very critical to prevent late sequelae following bone and joint infections and even death. The aim of this study was therefore to detect *K. kingae* infection in young children in Accra, Ghana. This study provides baseline information and aids in creating the awareness of *K. kingae* in Ghana.

## Methods

**Study design:** a cross-sectional study design was used.

**Study sites:** the study was conducted at the Greater Accra Regional Hospital (GARH), Trust Mother and Child Hospital (Trust Hospital) and Princess Marie Louise Hospital (PML), all in Accra. The PML is a quasi-private children hospital with a high attendance of children from the catchment area of Accra. The Trust Mother and Child Hospital, a satellite of the Trust Hospital, also has high attendance of children. GARH receives a lot of referral cases for children from other hospitals and clinics in Accra.

**Study subjects:** children 6 months to 4 years with a febrile illness or suspected localized bacterial infections (such as arthritis (joint pain) and osteomyelitis), limp, or restricted limb movement, with no history of trauma, directed by the clinician to undergo routine blood culture tests, were recruited. Children 4 years and below who reported with a febrile illness but who would not undergo routine blood culture tests were excluded.

**Ethical considerations:** this study was approved by the Ethics and Protocol Review Committee of the School of Biomedical and Allied Health Sciences, College of Health Sciences, University of Ghana, Korle-bu, Accra. Approval was also sought from the management of the Trust Hospital. For PML and GARH, approval was sought from the Regional Health Directorate of Greater Accra Region. Parents and/or guardians of all the children completed written informed consent forms.

**Sample collection:** between 1-3 ml of blood was taken from the antecubital vein of each child through venepuncture and dispensed into paediatric blood culture vials and universal blood culture bottles with brain heart infusion medium (BHI), using standard aseptic techniques [19, 20]. A drop of each blood sample (40 μl) was spotted on Whatman TM FTA cards (Whatman TM Inc., Brentford, UK) and air-dried. The Whatman TM FTA spotted cards were then kept in separate clean zipper bags for PCR analysis. The sample collection was done over five months, from April to August 2016.

### Culturing

#### BACTEC system

The blood culture vials were sent to the Clinical Microbiology Department of the Trust Hospital. The bottles were entered into the BACTEC™ FX40 system (Becton Dickinson, Cockeysville, MD, USA) for incubation at 37°C. The BACTEC™ FX40 system reports positive blood cultures (cultures with bacteria present, thus indicating the patient is “bacteremic”). Cultures were monitored for five days, after which negative vials were removed. Positive vials were retrieved and Gram stained for a rapid general morphologic identification of the bacteria. The blood was then sub-cultured onto blood agar, MacConkey agar and chocolate agar plates. The plates were incubated at 37^o^C for 24 hours and isolates subjected to susceptibility testing and biochemical tests. The plates were examined for ß-haemolysis, corrosion marks on the agar surface and failure to grow on MacConkey medium. For Gram-negative isolates, biochemical tests were carried out and examined, looking out for non-motility, weak oxidase reaction, a negative catalase reaction, urease and indole tests reactions, and with rare exceptions, production of acid from glucose and maltose [21].

#### Brain heart infusion (BHI) broth

The blood samples dispensed into BHI broth were incubated at 37°C for 24 hours. After overnight incubation, the samples were sub-cultured onto blood agar, MacConkey agar and chocolate agar plates and incubated again at 37°C in the incubator for 24 hours for bacterial growth. Gram stain was also performed on the isolates from the agar plates. Biochemical tests were performed on Gram-negative isolates. The samples were incubated again to enable slow growing bacteria to grow. After five days of incubation, the BHI sample was again sub-cultured, Gram staining was performed for a presumptive general morphologic identification of the bacteria. The plates were examined as done for the BACTEC cultures.

### PCR detection of *K. kingae*

#### DNA extraction

Dried blood spots on the filter paper were cut into small pieces with scissors and transferred into 1.5-ml microtubes. For lysis, a modified salting out DNA extraction protocol with several modifications based on Asadzaheh, Javanmard [22] was used. The extraction was carried out by using TNES buffer (10 mM Tris-HCl (pH 7.5), 400 mM NaCl, 100 Mm EDTA, 0.60% SDS) protocol. For lysis, 200 µl TNES digestion buffer was added to the filter papers, followed by adding 6 µl of proteinase K (10 mg/ml) to each tube and incubation for 4 hours in a heat block (Thermo Block TDB-120, Warren, United States of America) at 56°C. Samples were retrieved, 100 μl 5M NaCl was added to each tube and mixed briefly. The contents were placed in a freezer for 5 minutes, 20 μl protein precipitator was added to each tube, then the tubes were placed in a freezer for 10 minutes and spun in a centrifuge at 15000 rpm for 10 minutes. The supernatants were transferred into new 1.5 ml Eppendorf tubes, and 400 µl 100% ethanol added to each tube, then rocked gently back and forth. Deoxyribonucleic acid (DNA) precipitation was visible at this moment. Samples were stored in a -21^o^C freezer for 30 minutes, retrieved, allowed to thaw, and spun down at 15000 rpm for 10 minutes. The absolute ethanol was carefully poured off the pellet, 200 µl 70% ethanol added and spun again at, 15000 rpm for 5 minutes. The 70% ethanol was poured off after spinning, and tubes were blotted and air-dried. The formed pellets were finally re-suspended in 40 μl TE buffer (10 mM Tris-HCl, pH 8.0; 1 mM EDTA, pH 8.0.) and stored at -21°C.

### PCR analysis

Extracted DNA was initially subjected to PCR using universal prokaryotic primers p91E [5´-GGAATTCAAA (G/T) GAATTGACGGGGGC-3´] and p13B [5´-CGGGATCCCAGGCCCGGGAACGTATTCAC-3´] [23] to amplify a 475-bp fragment of the 16S rDNA gene. PCR was performed using OneTaq® Quick-Load® 2X Master Mix with standard buffer (NEB) and 0.25 uM of each primer. Conditions for amplification were an initial denaturation at 94°C for 4 minutes, followed by 40 cycles of 94^o^C for 1 minute, 56°C for 1 minute and 68°C for 2 minutes, with a final extension at 68^o^C for 10 minutes. Samples positive for 16S bacterial sequences were selected and used for another round of PCR using rtxA toxin gene primers of *K. kingae* as described previously [24]. Amplification was carried out with the primers F2-seq-rtxC [5´-GCCGAATGGGAAGATTTCTG-3´] and R2-seq-rtxA [5´-GCATTCATAAACGCCAACG-3´]. Conditions for amplification were an initial denaturation at 94°C for 4 minutes, followed by 40 cycles of 94°C for 1 minute, 56^o^C for 30 seconds and 68^o^C for 1 minute, with a final extension at 68°C for 10 minutes. All PCR reactions were carried out in a SEEAMP™ SCE1000 thermal cycler (Seegene Inc., Seoul, Korea). All amplification products were visualized under short wavelength U.V. after migration in 2.0% ethidium bromide-stained agarose gels and photographed using a Kodak EDAS 290 (New York, USA) gel documentation system. The sizes of the PCR products were estimated by comparing with the mobility of a standard 100 bp DNA ladder (New England Biolabs Inc., Ipswich, MA, USA).

### Data analysis

Data collected was entered and analysed using SPSS version 24.0 (IBM Corp, N.Y, USA) to obtain descriptive data. Relevant tables and figures were created from the data to allow for easy analysis and interpretation. Frequency and percentage were used to determine the prevalence of *K. kingae* among the children. Continuous variables such as age were presented in ranges.

## Results

**Demographic data:** a total of 232 children, 114 (49.1%) males and 118 (50.9%) females, were recruited for the study. Their ages ranged from 6 months to 48 months with a mean age of 20.10 ± 12.57 months.

**Figure 1:**
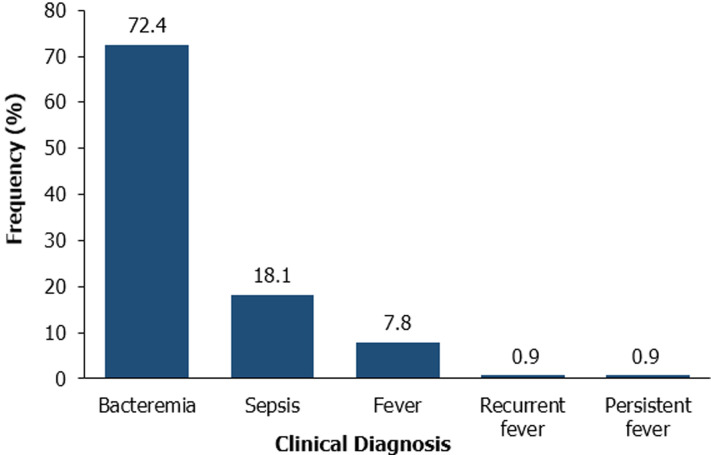
clinical diagnosis of the children by the clinicians

**Clinical diagnosis by clinicians:** the clinical diagnosis of the children by the clinicians is shown in Figure 1. Bacteremia (72.4%) was the highest diagnosis. Recurrent fever and persistent fever were the least (~1% each) diagnosis. Clinical diagnosis for any condition was highest in the 12-24 months age group (Table 1).

**Table 1:** clinical diagnosis in relation to age group

Age Group (Months)	Clinical Diagnosis (n, % within diagnosis)					Total
	Bacteremia	Sepsis	Fever	Recurrent fever	Persistent fever	
<12	46 (27.4)	10 (23.8)	8 (44.4)	1 (50.0)	0 (0.0)	65 (28.0)
12-24	79 (47.0)	15 (35.7)	10 (55.6)	1 (50.0)	2 (100.0)	107 (46.1)
25-36	27 (16.1)	11 (26.2)	0 (0.0)	0 (0.0)	0 (0.0)	38 (16.4)
37-48	16 (9.5)	6 (14.3)	0 (0.0)	0 (0.0)	0 (0.0)	22 (9.5)
**Total**	168 (100.0)	42 (100.0)	18 (100.0)	2 (100.0)	2 (100.0)	232 (100.0)

**Culture tests:** only seven (3.0%) out of the 232 culture tests were positive, showing bacterial growth. The rest showed no bacterial growth and 2 (0.9%) no bacterial growth but had contamination.

**Culture results in relation to clinical diagnosis:** Table 2 shows culture results in relation to clinical diagnosis. Out of the 232 samples, 168 (72.4%) were diagnosed as bacteremia of which 162 (96.4%) were culture-negative, 4 (2.4%) were culture positive and 2 (1.2%) were contamination. Of the 42 diagnosed as sepsis cases, 3 (7.14%) were positives for culture. The diagnosis for recurrent fever and persistent fever showed no bacterial growth.

**Table 2:** comparison of culture results and clinical diagnosis

Culture results	Clinical diagnosis (n, % within diagnosis)					Total
	Bacteremia	Sepsis	Fever	Recurrent fever	Persistent fever	
Bacterial growth	4 (2.4)	3 (7.1)	0 (0.0)	0 (0.0)	0 (0.0)	7 (3.0)
No bacterial growth	162 (96.4)	39 (92.9)	18 (100.0)	2 (100.0)	2 (100.0)	223 (96.1)
Contamination	2 (1.2)	0 (0.0)	0 (0.0)	0 (0.0)	0 (0.0)	2 (0.9)
**Total**	168 (100.0)	42 (100.0)	18 (100.0)	2 (100.0)	2 (100.0)	232 (100.0)

### PCR results

**Figure 2:**
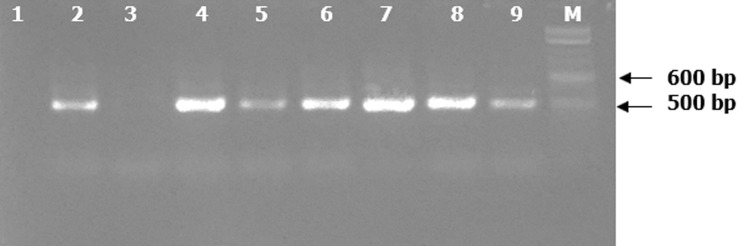
ethidium bromide-stained 2.0% agarose gel electrophoregram of amplified 16S rDNA fragments (475 bp) from samples using the p91E and p13B primers; Lane M = 100 bp marker (New England Biolabs Inc., Ipswich, MA, USA). Lanes 2 and 4 - 9 = PCR positives for 16S rDNA genes; Lanes 1 and 3 = PCR negatives for16S rDNA genes

**Detection of bacteria using universal prokaryotic primers:** bacterial 16S rDNA fragments of the predicted sizes (Figure 2) were successfully amplified in 223 (96.1%) out of 232 samples using the universal prokaryotic p91E and p13B primers.

**Figure 3:**
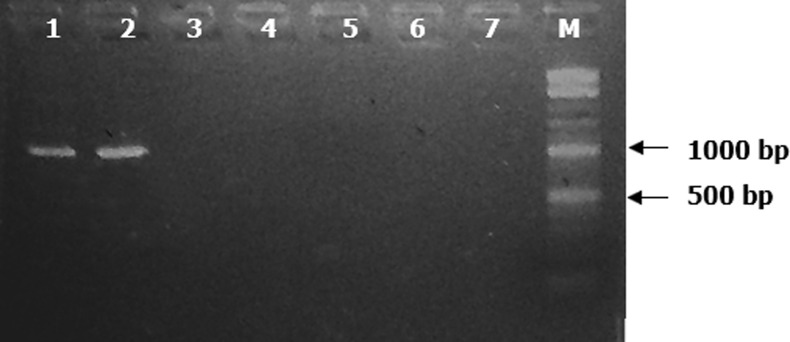
ethidium bromide-stained 2.0% agarose gel electrophoregram of amplified *K. kingae* rtxA toxin gene fragments (1198 bp) using the rtxA primers; Lane 8= 100 bp marker (New England Biolabs Inc., Ipswich, MA, USA); Lanes 1 and 2 = PCR positive for *K. kingae*; Lanes 3 - 7 = PCR negative for *K. kingae*

**Detection of *K. kingae* using rtxA toxin gene primers:** twelve (5.4%) samples yielded DNA fragments of the predicted size (approximately 1198 bp) (Figure 3) after screening of the 223 PCR positive samples for the 16S rDNA primers for *K. kingae* using the rtxA toxin gene primers. The 12 *K. kingae* PCR positives were detected in the children across all the age groups though more positives were within 12 -24 months and 25-36 months (Table 3) age groups.

**Table 3:** age group and *K. kingae* PCR results

Age (months)	*Kingella* PCR (n, % within PCR)		Total
	Positive	Negative	
<12	2 (16.7)	60 (28.4)	62 (28.7)
12 -24	4 (33.3)	99 (46.90)	103 (46.2)
25-36	4 (33.3)	33 (15.6)	37 (16.6)
37 -48	2 (16.7)	19 (9.0)	21 (9.4)
**Total**	12 (100.0)	211 (100.0)	223 (100.0)

**Bacteria rDNA PCR in relation to culture results:** out of the 223 positive 16S rDNA samples, 214 (96.0%) were culture-negative, 7 (3.1%) were positive for culture and 2 (0.9%) showed contamination.

**Culture results in relation to *K. kingae* PCR results:** comparison of culture results to *K. kingae* PCR results showed that out of the 12 positive *K. kingae* detected by PCR, 1 (8.3%) sample was culture positive and 11 (91.7%) samples were negative by the culture methods.

**Clinical diagnosis in relation *K. kingae* PCR:** Table 4 shows the comparison between the clinical diagnosis and the *K. kingae* PCR results. The 12 *K. kingae* PCR positive samples were all bacteremia cases.

**Table 4:** comparison of clinical diagnosis and *K. kingae* PCR results

Clinical diagnosis	*Kingella* PCR (n, % within PCR)		Total
	Positive	Negative	
Bacteremia	12 (100.0)	153 (72.5)	165 (74.0)
Sepsis	0 (0.0)	38 (18.0)	38 (17.0)
Fever	0 (0.0)	16 (7.6)	16 (7.2)
Recurrent fever	0 (0.0)	2 (0.9)	2 (0.9)
Persistent fever	0 (0.0)	2 (0.9)	2 (0.9)
**Total**	12 (100.0)	211 (100.0)	223 (100.0)

## Discussion

In this study, *K. kingae* detection by culture, broad range PCR and the toxin specific rtxA genes were investigated. *Kingella kingae* is a common etiology of paediatric bacteremia [2, 10]. In this study, bacteremia (72.4%) was the highest diagnosis by clinicians. However, blood culture showed bacterial growth in 7 (3.1%) samples, whereas the universal 16S rDNA detected 223 (96.1%) samples containing bacterial DNA. Detection of bacteria in a patient´s blood has diagnostic and prognostic importance, and blood cultures are essential in the diagnosis and treatment of the etiologic agents of bloodstream infections. Culturing of bacteria is the gold standard for the detection of bloodstream pathogens. Although it allows bacteria to be identified and their susceptibility profiles to be tested, it presents several limitations such as not being rapid because detection of bacterial growth requires approximately 12 - 48 h or more with fastidious bacterial [25]. Culturing also has low sensitivity for previous antibiotic treatment and/or low bacterial concentrations, due to the smaller blood sampled from paediatric patients than from adults [26, 27]. *Kingella kingae* may also appear Gram-positive on staining (Brujin *et al*., 2000: Murray *et al*., 2003). In addition, blood culture may allow the growth of a small quantity of bacteria potentially considered contaminants. However, many of these issues can be overcome by using nucleic acid amplification assays (NAAAs) like PCR which also reduces the time needed for bacterial identification from 3-4 days to a few hours. Using NAAAS also enables exact identification without suffering the bacteriostatic effect of antimicrobial therapy (Rothman *et al*., 2002: Ceroni *et al*., 2012). Some studies have reported detecting *K. kingae* from culture-negative specimens by using broad range PCR amplification (Moumile *et al*., 2003; Verdier *et al*., 2005; Matta *et al*., 2007; Chometon *et al*., 2007; Rosey *et al*., 2007). For example, a comparative study by Rosey *et al*. (2007) detected 15 additional cases using a combination of conventional and real-time PCR with broad-spectrum primers as against inoculated blood culture vials and seeded samples of synovial fluid onto solid medium which resulted in the isolation of *K. kingae* in 6 of 94 (6.4%) children. Using primers targeting the conserved 16S rDNA of bacteria serves as a control to monitor the extraction and absence of PCR inhibitors. PCR-based assays that amplify the 16S rRNA gene results in a 200% improvement in the diagnosis of the organism compared to culture [3]. However, several advantages of the PCR-based detection have a potential limitation of the detection of DNA from dead microorganisms, resulting in clinically false-positive results. These tests rely on all bacteria' having these 16S rDNA genes, though different bacterial species possess different numbers of copies, tied to their rate of growth [28].

Disease caused by *K. kingae*, believed to begin with asymptomatic colonization of the respiratory tract by disrupting the oropharyngeal epithelium which facilitates its entry into the bloodstream and damage to deeper tissues, was related to the production of a potent cytotoxin (RTX) which has a disease-promoting effect [29]. The results obtained by this study revealed that the 223 samples that underwent PCR testing for K. kingae using the set of primers specific for rtxA gene had 12 (5.4%) being K. kingae positive;11 (91.7%) of these were culture-negative samples. A molecular study by Ceroni *et al*. (20^10^) reported that 82% of the joint or bone aspirates of children younger than 4 years with osteoarticular infection were positive for *K. kingae*. PCR enhances detection of *K. kingae* in samples compared with routine cultures and blood culture vials [10]. Reported cases in Israel indicate that 40-50% of culture-negative septic arthritis cases in children might be attributable to *K. kingae* [30]. These results clearly show the superiority of molecular methods in diagnosing culture-negative paediatric arthritis (Rosey *et al*., 2007). The 12 *Kingella kingae* positive samples were all bacteremia cases by clinical diagnosis. This was not surprising as *K. kingae* occult bacteremia is the second most common presentation of *K. kingae* disease in children [9, 31]. Dubnov-Raz, Ephros (9) in their study revealed 38.8 ± 0.8°C as the maximal temperature measured in children with this condition, half with a body temperature of 39°C and one-third with a leucocyte count >15,000 WBC/ml. Thus, relying on guidelines based on the height of fever and leukocyte count results for obtaining blood cultures for management of young febrile children with no apparent focus [32], may not be sensitive enough for detecting *K. kingae* bacteremia.

As an oropharyngeal colonizer, *K. kingae* is transmitted by respiratory secretions, saliva, and potentially oral contact with contaminated objects. Asymptomatic colonization of the upper respiratory tract by *K. kingae* is found in children who acquire the infectious agent after six months of life [10]; subsequently, the incidence of colonization decreases to 10-12% until the end of the second year before gradually declining to low levels in older children and adults. This suggests that the disappearance of vertically transmitted immunity and the greater socialisation of children aged over six months increase the risk of colonization, whereas progressive immunological maturation leads to acquiring sufficient immunity to eradicate the organism from the pharynx in older people. These agree with the results of the present study, where more of the positive *K. kingae* were detected at age 1-3 years. In several studies, almost 90% of the reported cases have occurred in children aged < 5 years, and 60% in those aged < 2 years [33, 34]. Data over a 23-year period from the Soroka University Medical Centre in southern Israel indicated that age distribution of affected children show that occurrence of disease below the age of 6 months is exceptional. Cases rapidly accumulate thereafter, reaching a peak in children aged 6-11 months [9].

## Conclusion

*Kingella kingae* was not detected by microbiological means but was detected only by PCR assay specific for the *K. kingae* RTX toxin. Most PCR positives were within the 12-36 months age group and all bacteremia cases by clinical diagnosis. Hence, *K. kingae* may be a potential cause of bacteremia and hence febrile illness in young children living in Accra, Ghana.

### What is known about this topic

- Kingella kingae is a common etiology of paediatric bacteremia;
- Data on K. kingae infections from sub-Saharan Africa are scarce.

### What this study adds

- First report of K. kingae infection in Accra, Ghana;
- Kingella kingae may be a potential cause of bacteremia in young children in Accra, Ghana.
